# Effect of simultaneous presence of anti-blood group A/B and -HLA antibodies on clinical outcomes in kidney transplantation across positive crossmatch: a nationwide cohort study

**DOI:** 10.1038/s41598-019-54397-3

**Published:** 2019-12-03

**Authors:** Hyunwook Kwon, Jee Yeon Kim, Dong Hyun Kim, Youngmin Ko, Ji Yoon Choi, Sung Shin, Joo Hee Jung, Young Hoon Kim, Duck Jong Han, The Korean Organ Transplantation Registry Study Group, Curie Ahn, Dong Wan Chae, Jaeseok Yang, Bum Soon Choi, Cheol Woong Jung, Myung Soo Kim, Oh Jung Kwon, Jae Berm Park, Yeong Hoon Kim, SooJinNa Choi, Seung Yeup Han, Sang Ho Lee, Kyung Hwan Jeong, Seung Jung Kim, Jin Seok Jeon, Yeon Ho Park, Young Nam Roh, Jeong Joon Lee, Kang Wook Lee, Seung Yeup Han, Chan Duck Kim, Jong Won Park, Joong Kyung Kim, Dong Ryeol Lee, Dong Won Lee, Eun Young Seong, Jin Min Kong, Hong Rae Cho, Sung Kwang Park, Sam Yeol Lee, Jung Hwan Park

**Affiliations:** 1 Division of Kidney and Pancreas Transplantation, Department of Surgery, Asan Medical Center, University of Ulsan College of Medicine, Seoul, Korea; 2 Transplantation Center, Seoul National University Hospital, Seoul, Korea; 3 Department of Internal Medicine, The Catholic University of Korea, Seoul, Korea; 4 Department of Surgery, Korea University Anam Hospital, Seoul, Korea; 5 Department of Surgery, Severance Hospital, Yonsei University College of Medicine, Seoul, South Korea; 6 Department of Surgery, Hanyang University Hospital, Seoul, South Korea; 7 Department of Surgery, Samsung Medical Center, Sungkyunkwan University School of Medicine, Seoul, South Korea; 8 Organ Transplantation Center, Busan Paik Hospital, Inje University College of Medicine, Seoul, South Korea; 9 Department of Surgery, Chonnam National University, Gwangju, South Korea; 10 Department of Internal Medicine, Keimyung University School of Medicine, Daegu, South Korea; 11 Department of Internal Medicine, Kyunghee University College of Medicine, Seoul, South Korea; 12 Department of Internal Medicine, Ewha Womans University Medical Center, Seoul, South Korea; 13 Department of Internal Medicine, Soon Chun Hyang University Hospital, Seoul, South Korea; 14 Department of Surgery, Gachon University Gil Medical Center, Seongnam, South Korea; 15 Organ Transplantation Center, Inje University Ilsan Paik Hospital, Goyang, South Korea; 16 Department of Surgery, CHA Bundang Medical Center, CHA university, Seongnam, South Korea; 17 Department of Internal Medicine, Chungnam National University Hospital, Daejeon, South Korea; 18 Department of Internal Medicine, Keimyung University Dongsan Medical Center, Daegu, South Korea; 19 Department of Internal Medicine, Kyungpook National University Hospital, Daegu, South Korea; 20 Department of Internal Medicine, Yeungnam University Hospital, Gyeongsan, South Korea; 21 Department of Internal Medicine, Bong Seng Memorial Hospital, Seoul, South Korea; 22 Department of Internal Medicine, Maryknoll medical center, Maryknoll, Australia; 23 Department of Internal Medicine, Pusan National University Hospital, Busan, South Korea; 24 Department of Internal Medicine, BHS Han Seo Hospital, Busan, South Korea; 25 Department of Surgery, Ulsan University Hospital, Ulsa, South Korea; 26 Department of Internal Medicine, Chonbuk National University Hospital, Jeonju, South Korea; 27 Hallym University Medical Center, Chuncheon, South Korea; 28 Department of Internal Medicine, Konkuk University Medical Center, Chungju, South Korea

**Keywords:** Allotransplantation, Immune tolerance

## Abstract

ABO-incompatible (ABOi) and positive crossmatch (XM) kidney transplantation (KT) have been considered immunologically challenging. The present study analyzed the clinical outcomes in XM positive KT based on ABO incompatibility. We used data from the Korea Organ Transplantation Registry, a nationwide database, and a single-center registry. A total of 263 patients with positive XM were divided into an ABO compatible (ABOc) & XM positive (ABOc/XM+, n = 176) group and an ABOi & XM positive (ABOi/XM+, n = 87) group. The overall rejection rate one year after KT was significantly higher in the ABOi/XM+ group than in the ABOc/XM+ group (*P* < 0.01). A total of four mortalities occurred, all in the ABOi/XM+ patients (*P* < 0.01). There were no differences in surgical complications or the occurrence of infection-related complications, including BK virus nephropathy. Multivariate analysis indicated that female vs. male (odds ratio (OR), 2.27; *P* = 0.03), DSA class I (MFI/1000) (OR, 1.10; *P* = 0.03), DSA class II (MFI/1000) (OR, 1.10; *P* < 0.01), and ABOi & XM+ status (OR, 2.38; *P* < 0.01) were significant risk factors for acute rejection during the year after transplantation. Overall graft survival was inferior in ABOi/XM+ patients than in ABOc/XM+ patients (*P* = 0.02). ABO incompatibility in XM-positive KT patients was found to be a significant risk factor for the development of rejection within one year after transplantation as well as for long-term graft survival. The anti-blood group A, B and anti-HLA antibodies may show synergistic activity.

## Introduction

There have been efforts to extend the donor pool for kidney transplantation (KT), including transplants across anti-blood group A, B and the human leukocyte antigen (HLA) antibody (Ab) group^1,2^. Development of desensitization treatment and Ab monitoring methods have made KT possible in patients who were ABO or HLA incompatible^1,3–6^. Recent reports demonstrated that ABO-incompatible (ABOi) KT patients have graft survival (GS) rates similar to those with ABO-compatible (ABOc) KT, although the rates of Ab-mediated rejection (AMR) and infectious complications were more common after ABOi KT^2,7–10^. HLA incompatible (HLAi) KT, especially in recipients with positive complement-dependent cytotoxicity (CDC) or flow-cytometric (FC) crossmatch (XM), has a much higher rate of AMR than HLA compatible (HLAc) KT^1,6^. However, transplant across the HLA barrier with desensitization treatment is believed to contribute to improving the patient survival rate rather than waiting for an HLA-c transplant^11^.

The desensitization protocols for both ABOi and HLAi KT are similar while the intensity and monitoring method differs^1,2,5,6^. New immunosuppressive medication, plasmapheresis (PP), and intravenous immunoglobulin (IVIG) have promising clinical outcomes allowing immunologically high-risk patients to receive a transplant^12^. KTs in recipients who have both anti-blood group A, B and HLA Abs are considered at high risk for immediate rejection, as seen in classic studies which became relatively popular these days^13^. There have been a few studies that revealed the clinical outcomes of transplant in patients with both ABOi and HLAi^4,5,9,13–15^. There have been no prior reports on the synergistic effects of anti-blood group A, B and HLA abs in such patients, although there was a report indicating that patients who had both barriers may need more interventions during the peri-operative period^13^. However, these studies had only a small sample size not sufficient to have statistical power, and they included patients who had a low titer of anti-HLA donor-specific antibody (DSA).

In this study, we utilized combined data from the Korea Organ Transplantation Registry (KOTRY), an established nationwide database, and the single-center registry of Asan Medical Center (AMC), to secure the number of patients included. To investigate the impact of anti-blood group A, B Ab on HLAi KT in immunologically high-risk patients, the present study analyzed the clinical outcomes in XM positive KT based on ABO incompatibility.

## Materials and Methods

### Patients

**Figure 1:**
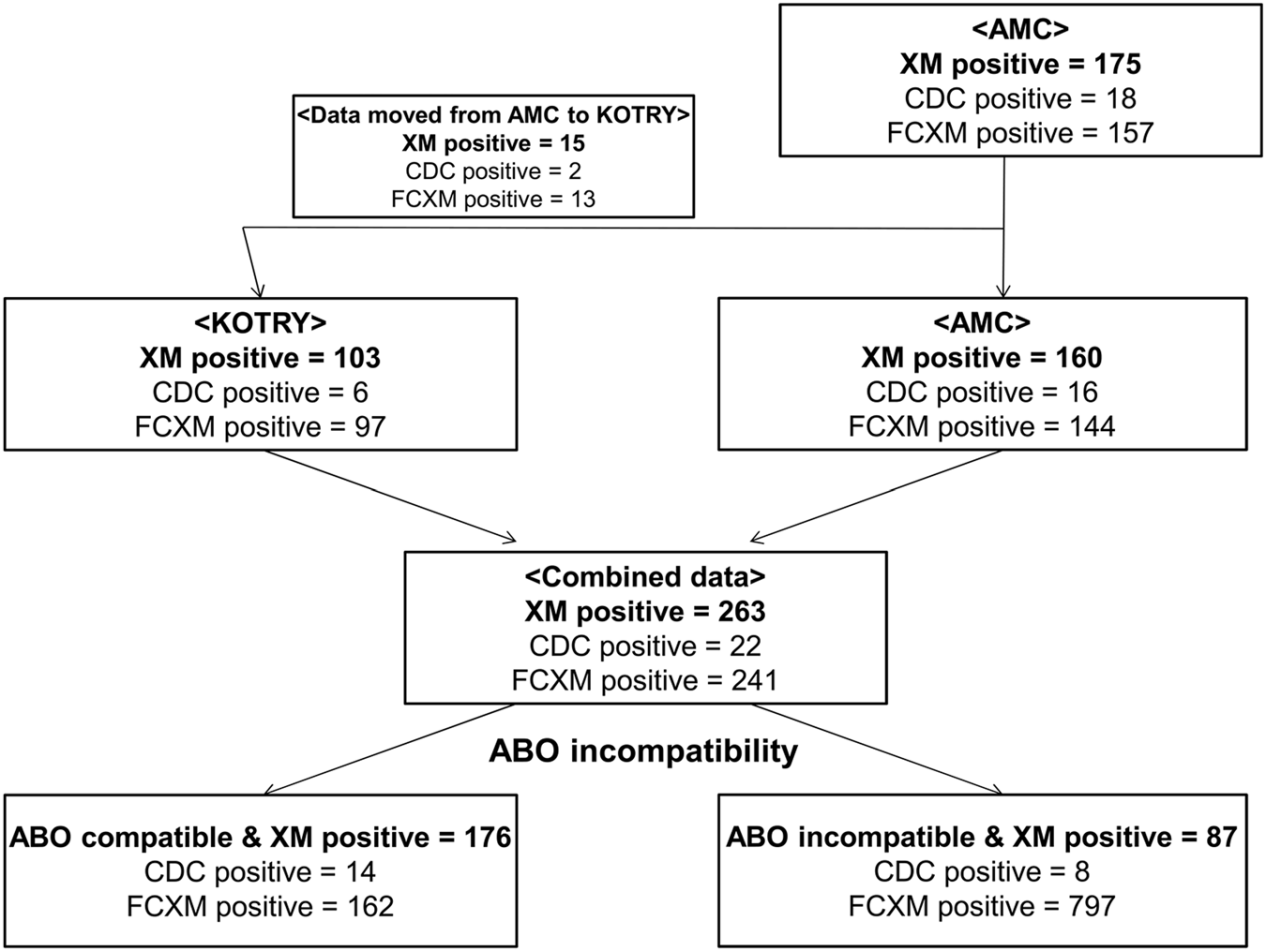
Flow chart of patient inclusion.

This retrospective study used extracted data from 46 KOTRY participating medical centers in South Korea as well as AMC transplant center’s registry. We included patients who underwent CDC or FCXM positive KT from living donors between January 2014 and December 2016 according to the KOTRY database and between January 2009 and December 2016 from AMC. After excluding 15 cases of XM-positive KT that were input from AMC to the KOTRY data, 103 KOTRY patients and 160 patients from AMC data were combined for this analysis. A total of 263 patients, including 22 patients with positive CDC and 241 with positive FCXM, were divided into an ABOc & XM positive (ABOc/XM+, n = 176) group and an ABOi & XM positive (ABOi/XM+, n = 87) group (Fig. 1). XM positive transplant was defined as KT in patients with positive CDC and/or positive FCXM. The medical records were reviewed after receiving informed consent^16^. The clinical and research activities being reported are consistent with the Principles of the Declaration of Istanbul, as outlined in the Declaration of Istanbul on Organ Trafficking and Transplant Tourism. NO organs/tissues were procured from prisoners. Organs/tissues were procured only at registered institutions with The Korean Network for Organ Sharing, which is a nationwide system of deceased donor detection and distribution^17^. The Asan Medical Center institutional review board (IRB organizations’ IORG number: IORG0009892/Federal wide assurance number: 00005513) approved this study (AMC IRB number of this study 2013–0319).

### Immunologic tests and definition

HLA typing assays for HLA-A, -B and for DRB1 by Sequence-Based Typing detected HLA mismatches. The DSA was measured using HLA class I and II single antigen bead (SAB). A Luminex system (One Lambda) detected fluorescence, expressed as the mean fluorescence intensity (MFI) of each SAB. The CDC test was performed using T-cells from the donor and anti-human globulin (AHG) added sera derived from recipients. The cut value of positive FCXM in each medical center was slightly different. In general, T-cell and B-cell FCXM were considered positive when the ratio of the mean MFI to the control MFI exceeded 2.0 and 2.5 (77 for T-cell and 101 for B-cell FCXM of median channel shift on a 1024 scale), respectively. The anti‐A or ‐B titer was measured by the standard tube method using saline for IgM and indirect Coombs’ testing for IgG. Patient survival (PS) and graft survival (GS) were defined as the time from KT to patient death and the time from transplantation to return to dialysis, graft loss, or the last follow-up date with a functioning graft. Protocol biopsies were not routinely performed. Acute rejection (AR) was diagnosed pathologically according to the Banff criteria^18^. Clinical rejection without indication biopsy was not included as acute rejection

### Desensitization and immunosuppression

**Figure 2:**
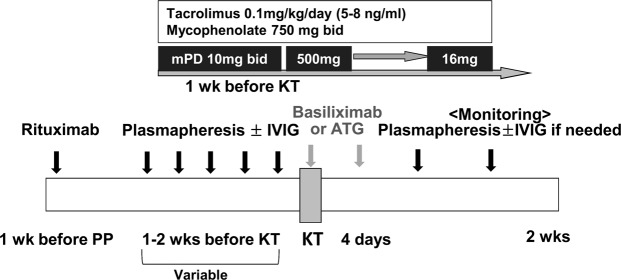
Desensitization protocol for patients undergoing crossmatch-positive and ABO-incompatible kidney transplantation.

Highly uniform desensitization protocols have been used across the medical centers in ABOi and XM positive KT (Fig. 2)^19^. A single dose of rituximab (anti-CD20 monoclonal Ab) (200–500 mg) was used from 1 week to 4 weeks before transplantation in both groups. PP with or without IVIG administration followed until patients reached the treatment target. The goal of desensitization in XM-positive KT was a negative conversion of AHG-CDC and T-cell FCXM. In ABOi KT, the therapeutic target was slightly different according to the medical centers and the range of the anti-blood group A, B titer was from 4 to 16. In case of a rebound in the anti-blood group A, B titer during first 2 weeks after transplantation, additional PP treatments were performed until the titer was within each center’s target range. The anti-IL-2 receptor Ab (basiliximab, once on day 0 and once on day 4) or anti‐thymocyte globulin (ATG, total dose 2.8–6.2 mg/kg, administered on day 0 and postoperatively) was used as an induction regimen, according to each center’s protocols. For maintenance immunosuppressants, a calcineurin inhibitor (tacrolimus or cyclosporine), mycophenolic acid, and a corticosteroid were used.

### Statistics

Categorical variables presented as counts and percentages were compared using the chi-square test or Fisher’s exact test, whichever was appropriate. Continuous variables presented as means and standard deviations and were compared using the Student’s *t-*test. The GS and rejection-free graft survival (RFGS) rates were evaluated by using the Kaplan–Meier method and were compared using the log-rank test. The risk factors for AR during the first year after transplantation were assessed using logistic regression analysis. Variables showing significance with a *P-*value of 0.1 in the univariate analysis were introduced into multivariate analysis. A *P-*value of <0.05 was considered statistically significant, and all statistical analyses were performed using SPSS version 18.0 (SPSS Inc., Chicago, IL, USA).

## Results

### Patient demographics and clinical characteristics

The 263 patients who underwent XM positive KT and were enrolled in this study were divided into two groups according to ABO incompatibility with 176 (66.9%) in the ABOc/XM+ group and 87 (33.1%) in the ABOi/XM+ group. Their baseline characteristics are shown in Table 1; ABOi/XM+ patients were more likely to be female (*P* < 0.01) and have a lower degree of HLA class I PRA (*P* < 0.01). The proportion of positive CDC XM and T-flow in only positive recipients in FCXM was similar between both groups (*P* = 0.91). Otherwise, there was no significant difference between the two patient groups, including age, calcineurin inhibitors, induction therapy, duration of dialysis, and maximal DSA value.

**Table 1:** Baseline characteristics of the study population.

	^a^ABOc/XM+	^a^ABOi/XM+	*P*-value
Number of patients	176 (66.9)	87 (33.1)	
Mean age (years)	48.7 ± 11.9	51.4 ± 9.6	0.12
Female sex	46 (26.1)	42 (48.3)	<0.01
Body mass index (kg/m^2^)	22.2 ± 3.1	25.5 ± 13.5	0.06
**Calcineurin inhibitor**			
Prograf	151 (85.8)	74 (85.1)	0.87
Cyclosporin	25 (14.2)	13 (14.9)	
Induction			0.24
ATG	30 (17.0)	10 (11.5)	
Basiliximab	146 (83.0)	77 (88.5)	
Previous transplant	28 (25.9)	19 (21.8)	0.22
Duration of dialysis (months)	23.6 ± 36.3	29.8 ± 37.3	0.33
Cross-matching results			0.91
CDC positive	14 (8.0)	8 (9.2)	
FCXM positive	162 (92.0)	79 (90.8)	
T-flow only positive	83 (47.2)	39 (44.8)	
T- or B- flow positive	79 (44.9)	40 (46.0)	
PRA class I	42.3 ± 38.0	25.4 ± 34.1	<0.01
PRA class II	35.3 ± 38.4	30.2 ± 39.7	0.36
Maximal DSA (MFI)	7266 ± 4742	7187 ± 4923	0.94
DSA class I (MFI)	5486 ± 4356	3705 ± 4654	0.09
DSA class II (MFI)	5088 ± 5522	5209 ± 5576	0.93

Continuous data are presented as means ± standard deviations, whereas categorical data are presented as numbers (%).

Abbreviations: ATG, anti-thymocyte globulin; CDC, complement-dependent cytotoxicity; FCXM, flow-cytometric crossmatch; PRA, panel reactive antibody; DSA, donor specific antibody; MFI, mean fluorescence intensity.

^a^Crossmatch-positive (XM+) defined as FCXM-positive or CDC XM-positive; ABOc, ABO compatible; ABOi, ABO incompatible.

### Clinical outcomes at one year after transplantation

Assessments of the rejection profiles one year after KT showed that the overall rejection rate was significantly higher in the ABOi/XM+ group than in the ABOc/XM+ group, i.e., 25 patients (25.5%) vs. 21 patients (11.7%); *P* < 0.01. This finding was mainly due to the ABOi/XM+ group having a significantly higher incidence of AMR than the ABOc group (*P* = 0.01). However, there was no difference in the ACR rate at one year after transplant between the two groups (*P* = 0.17). In subjects with positive CDC, all five rejection episodes (62.5%) developed in 8 patients in the ABOi/XM+ group; all five of these cases were due to AMR. There was no episodes of rejection in 14 patients in the ABOc/XM+ group. In subjects with positive FCXM, the ABOi/XM+ group had a tendency without statistical significance toward higher overall rejection rate than the ABOc/XM+ group, i.e., 17 patients (21.5%) vs. 21 patients (13.0%); *P* = 0.09. All four cases of mortality occurred in the ABOi/XM+ group (*P* < 0.01). The four deaths were caused by pneumonia (two patients), myocardial infarction (one patient), and hypovolemic shock due to bleeding (one patient). There was no difference between the two groups in infectious complications such as bacterial infection and BK virus nephropathy (BKVN) (*P* = 0.14) as well as in surgical complications (*P* = 0.30; Table 2). Logistic regression analysis was performed to evaluate the risk factors associated with AR during the year after transplantation (Table 3). Univariate analysis showed that variables such as female gender, DSA class I (MFI/1000), DSA class II (MFI/1000), and both XM positivity and ABOi patients had statistical significance. After adjustment for these confounding factors, multivariate analysis indicated that female vs. male (odds ratio (OR) = 2.27; 95% confidence interval (CI), 1.10–4.72; *P* = 0.03), DSA class I (MFI/1000) (OR = 1.10; 95% CI, 1.01–1.20; *P* = 0.03), DSA class II (MFI/1000) (OR = 1.10; 95% CI = 1.03–1.18; *P* < 0.01), and both XM positivity and ABOi patients (OR = 2.38; 95% CI = 1.21–4.73; *P* < 0.01) were found to be significant risk factors for AR during the first year after transplantation.

**Table 2:** Clinical outcomes at one year after transplantation.

	^a^ABOc/XM+	^a^ABOi/XM+	*P*-value
Number of patients, XM+	176 (66.9)	87 (33.1)	
Overall rejection	21 (11.7)	25 (25.5)	<0.01
ACR only	5 (2.8)	6 (6.1)	0.17
AMR with or without ACR	16 (8.9)	19 (19.4)	0.01
Number of patients, CDC+	14 (63.6)	8 (36.4)	
Overall rejection	0 (0.0)	5 (62.5)	<0.01
ACR only	0 (0.0)	0 (0.0)	—
AMR with or without ACR	0 (0.0)	5 (62.5)	<0.01
Number of patients, FCXM+	162 (67.2)	79 (32.8)	
Overall rejection	21 (13.0)	17 (21.5)	0.09
ACR only	5 (3.1)	4 (5.1)	0.48
AMR with or without ACR	16 (9.9)	13 (16.5)	0.14
Mortality	0 (0.0)	4 (4.6)	<0.01
Bacterial infection			0.14
Urinary tract infection	39 (21.7)	12 (12.1)	
Pneumonia	14 (7.8)	7 (7.1)	
Biopsy proven BKVN	1 (0.6)	1 (1.1)	0.61
Surgical complications			0.30
Bleeding	10 (5.6)	2 (2.0)	
Urinary complications	3 (1.7)	3 (3.1)	

Values are presented as numbers of patients (%).

Abbreviations: ACR, acute cellular rejection; AMR, Acute antibody-mediated rejection; CDC, complement-dependent cytotoxicity; FCXM, flow-cytometric crossmatch; BKVN, BK virus nephropathy.

^a^Crossmatch-positive (XM+) defined as FCXM-positive and CDC XM-positive; ABOc, ABO compatible; ABOi, ABO incompatible.

**Table 3:** Factors associated with acute rejection during the first year after transplantation.

	Univariate analysis		Multivariate analysis	
	OR (95% CI)	*P*-value	OR (95% CI)	*P*-value
Female vs. male sex	1.91 (1.00–3.63)	0.05	2.27 (1.10–4.72)	0.03
Cyclosporin vs. Prograf	1.15 (0.45–2.91)	0.78		
Basiliximab vs. ATG	1.87 (0.84–4.16)	0.78		
CDC positive vs. FCXM positive	1.00 (0.39–2.57)	0.99		
PRA class I	1.00 (0.99–1.00)	0.24		
PRA class II	1.00 (0.99–1.02)	0.13		
DSA class I (MFI/1000)	1.08 (1.00–1.17)	0.05	1.10 (1.01–1.20)	0.03
DSA class II (MFI/1000)	1.10 (1.03–1.16)	<0.01	1.10 (1.03–1.18)	<0.01
^a^XM+ and ABOi vs. XM+ and ABOc	2.59 (1.36–4.93)	<0.01	2.38 (1.21–4.72)	0.01

Abbreviations: ATG, anti-thymocyte globulin; CDC, complement-dependent cytotoxicity; FCXM, flow-cytometric crossmatch; PRA, panel reactive antibody; DSA, donor-specific antibody; MFI, mean fluorescence intensity.

^a^Crossmatch-positive defined as FCXM-positive and CDC XM-positive; ABOc, ABO compatible; ABOi, ABO incompatible.

### Long-term clinical outcomes

**Figure 3:**
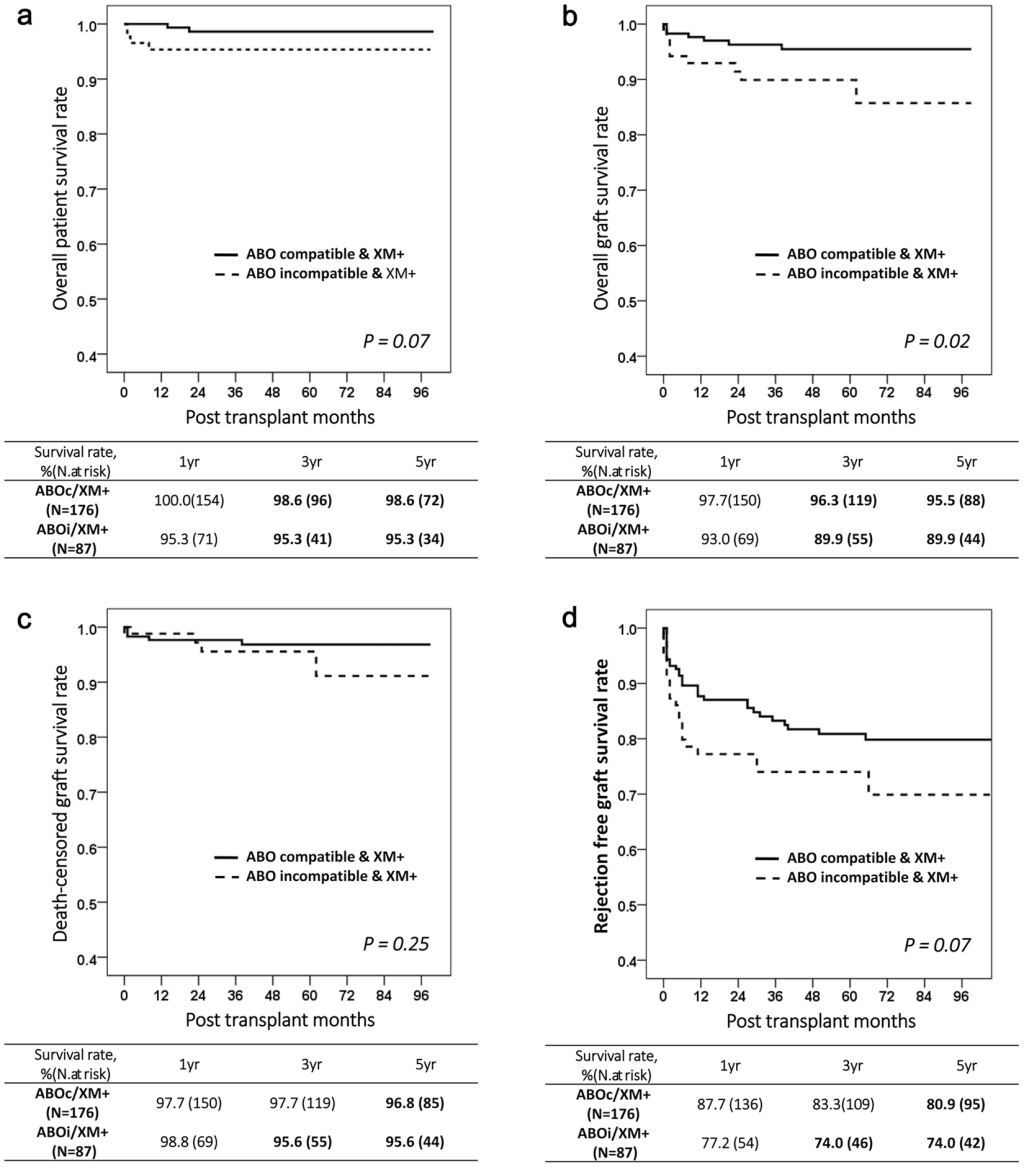
Long-term survival after kidney transplantation. (**a**) Overall patient survival. (**b**) Overall graft survival. (**c**) Death-censored graft survival (**d**) Rejection–free graft survival.

Kaplan–Meier analysis showed that the overall GS was inferior in ABOi/XM+ patients more so than in ABOc/XM+ patients (*P* = 0.02; Fig. 3b). The cumulative GS rates at one, three, and five years were, respectively, 97.7%, 96.3%, and 95.5% in the ABOc/XM+ group and 93.0%, 89.9%, and 89.9% in the ABOi/XM+ group. Although the PS (*P* = 0.07) and the RFGS (*P* = 0.07) in the ABOi/XM+ patients tended to be inferior to those in the ABOc/XM+ group, the difference had no statistical significance (Fig. 3a,d**)**. Death-censored graft survival (DCGS) did not show significant difference between two groups (*P* = 0.25; Fig. 3c**)**. The PS rate at one, three, and five years was 100.0%, 98.6%, and 98.6%, in the ABOc/XM+ group and 95.3%, 95.3%, and 95.3% in the ABOi/XM+ group. The RFGS rates at one, three, and five years were 87.7%, 83.3%, and 80.9% in the ABOc/XM+ group and 77.2%, 74.0%, and 74.0% in the ABOi/XM+ group, respectively.

## Discussion

This study indicates that ABOi KT in recipients with positive XM has a significantly inferior GS than those with ABOc KT, and which appears to be caused by higher rejection and mortality rates. The inferior one-year AR rate in patients with ABOi/XM+ and our multivariate analysis suggest that anti-blood group A, B and anti-HLA abs in KT across positive XM have a synergistic effect on the immunologic reaction after transplantation. This is the first published report to demonstrate the results of the immunologically challenged patient group, combined ABOi, and the XM positive subject, in relatively large numbers of patients more than recent studies have shown^4,5,9,13–15^. Although we are not able to provide the exact mechanism of how anti-blood group A, B and anti-HLA abs interact synergistically on a graft, novel information regarding the risk for ABOi and XM positive KT is introduced in this study.

The KOTRY study group previously reported that the ABOi and HLAi groups had a tendency to increase the incidence of rejection without statistical significance and failed to reveal the synergistic effect of ABO and HLA incompatibilities^5^. This study enrolled only 31 patients with ABOi and HLAi, and the HLAi group included XM negative patients with only DSA positive, as demonstrated by Luminex SAB. Similarly, Padmanabhan *et al*. indicated that ABOi- and XM-positive patients required more PP and IVIG treatment, both pre- and post-transplant and they showed a tendency to increase the incidence of late rejection, although the small group size of the XM positive group became a limitation for achieving statistical power^13^. Therefore, our study conducted an analysis, including combined recipient data of a single, large center registry with KOTRY data to ensure enough enrolled patients including immunologically high-risk patients who showed positive XM. Although data were not shown in the *Result* section, 296 patients who underwent ABOi and XM negative (ABOi/XM−) KT in KOTRY, during the same period as in this study, had a better 1-year AR rate (*P* < 0.01) and overall GS rate (*P* = 0.02) than ABOi/XM+ patients (Supplementary Table 1). However, our study showed that ABOc/XM+ versus ABOi/XM+ patients had a better 1-year AR and overall GS rate. Therefore, we concluded that ABO and HLA antibodies appeared to have a synergistic effect on clinical outcomes in KT.

We conducted univariate and multivariate logistic analysis for determining the risk factors associated with AR during the first year after KT in light of the larger group size than that of the remaining patients during long-term follow-up. In addition, the rejection episode primarily occurred early, especially within the first 30 days to one year after transplant, and patients who experienced early rejection were at high risk of developing late rejection^9^. Similarly, more than half of the transplant rejections, mainly AMR, was observed within one year after KT. The pattern of the Kaplan–Meier analysis graph for long-term RFGS and PS showed significant differences between the ABOc/XM+ and the ABOi/XM+ groups during the first year after transplant, followed by a similar pattern which resulted in failure to reach statistical significance. This finding suggests that the rejection and the PS rates of the first year after transplant determine the difference in the overall GS between the two groups.

The immunogenicity of ABO-i and HLA-i KT was different in terms of both the structure and antigenicity. The target epitopes of anti-blood group A, B were expressed on endothelial cells in the grafts, which differ from those on the erythrocyte membrane, and resided in a carbohydrate structure present in the form of glycoproteins^20^. This study suggests that circulating anti-blood group A, B Ab does not necessarily bind and react with ABO antigens expressed on endothelial graft cells. Takahashi believed that AMR due to anti-blood group A, B Ab is mainly caused by not natural but by de novo Ab, resulting occurrence especially two to seven days after transplant, which is called the “critical period”^21^. After stabilization of graft function, down-regulation of Ab production against the donor ABO antigen was acquired^22^. A phenomenon that the patients remain not rejected in the presence of a circulating antibody can be a possible theory for the relatively lower antigenicity of ABO-i KT than that of HLA-i KT^20,23,24^. Although DSA can exist without acute rejection after HLA-i KT, especially when its titer is low, even in those cases, subclinical rejection and chronic AMR frequently occurred^25^.

Numerous studies have reported the mechanism of accommodation after ABOi KT. Up-regulation of anti-inflammatory and anti-apoptotic genes, such as heme oxygenase-1, ERK inactivation resulting in complementary inhibitions by CD55 and CD 59, activation of the PI3K/cAMP-dependent PKA pathway, and endothelial chimerism, have all been suggested as possible explanations for accommodation^23,26–29^. However, there are still no confirmative studies demonstrating the interactions of anti- HLA and -blood group A, B Ab in the process of accommodation. Iwasaki *et al*. reported that ligation of anti-blood group A, B Ab-induced negative regulation of HLA-DR expression through inactivation of ERK and mTOR pathways^28^. This phenomenon may have a protective effect when anti-HLA ab is present at a low titer. Zhang *et al*. and the Iwasaki group reported that low titers of anti-HLA abs stimulate anti-apoptotic genes, thus leading to cell survival, while higher titers of HLA abs stimulate signaling pathways related to ab mediated activation of endothelial cells^23,30^.

Why ABOi KT in XM-positive recipients has a more substantial risk for rejection is speculative. One possible hypothesis is a depletion of the anti-apoptotic and protective process due to simultaneous exposure to both anti-HLA and -blood group A, B Ab. The comparable result of ABOi KT with that of ABOc KT induced by repair and an anti-inflammatory mechanism may not be maintained in the presence of a high level of anti-HLA Ab. The consuming repair process due to the anti-blood group A, B Ab may enhance toxicity by anti-HLA Ab. In the opposite sense, high titers of anti-HLA abs trigger activation of endothelial cells by upregulating a pro-inflammatory gene, such as ERK or the mTOR pathway, and thus causing ABOi KT to fail to achieve accommodation^23,27,30^. Our results support this hypothesis. Patients in the ABOi/XM+ group who developed rejection generally had a high level of DSA. In the CDC-positive group, all five AMRs occurred in ABOi/XM+ group. Multivariate analysis showed that high intensity of DSA class I and II were significant risk factors for AR. Another possible mechanism is the vulnerability of the immune system against pathogens due to blood type incompatibility. Sharif *et al*. reported that ABOi recipients were more likely to develop BKVN compared to HLAi patients^10^. Although our study did not show a difference in the incidence of BKVN in either group, it is possible that a more subclinical BK virus-related infection or an inflammatory change occurred in the ABOi group. In this study, more patients in the ABOi/XM+ group showed a higher incidence of infectious complications leading to their death. It appears that desensitization in the ABOi/XM+ group is more challenging than in the ABOc group or that ABO incompatibility alters the host defense mechanism, making it vulnerable to infection due to unrevealed reasons.

This study has several limitations. First, due to the utilization of data from a big registry, some variables were missing necessitating further analysis to interpret the clinical outcomes and each center had slightly different immunosuppressive protocols. For example, the number of PPs for desensitization, the titer of anti-blood group A, B Ab, and the number of patients who underwent post-operative PP were not available. In addition, the selection of induction agents and their doses were determined by each transplant center. However, the intensity of pre-transplant desensitization might be similar between our study groups because immunologic demographics, considered the primary determinant of desensitization treatment, showed no differences. Second, because of its retrospective design with data from multiple medical centers, there may have been information and selection biases. Third, the patients enrolled in this study consisted predominantly of Asian subjects. Therefore, our results may have a different aspect from that of other racial populations or for recipients in other medical circumstances.

In conclusion, ABO incompatibility in XM positive KT was a significant risk factor for the development of rejection within one year after transplantation. In addition, the long-term GS rate in ABOi and XM positive KT is inferior to that in ABOc and XM positive KT due to higher rejection and mortality rates. These findings suggest that anti-blood group A, B and anti-HLA abs may have a synergistic effect on the clinical outcomes after transplantation. Although there is an increasing unmet need for organ donation, consideration of balance between the risks and benefits of transplantation with personalized approach is essential before KT, especially in high-risk populations. Further research is needed to uncover the mechanism and evidence contributing to the synergistic effect of anti-blood group A, B and anti-HLA abs on the host immune system. Finding the answer to this question may contribute to our understanding of accommodation after transplant and overcome immunologic barriers in transplantation.

## Supplementary information

SUPPLEMENTARY TABLE 1
