# Supplementary material for: Effect of simultaneous presence of anti-blood group A/B and -HLA antibodies on clinical outcomes in kidney transplantation across positive crossmatch: a nationwide cohort study
Source: Sci Rep. 2019 Dec 3;9:18229. doi: 10.1038/s41598-019-54397-3 (PMC6890690; doi:10.1038/s41598-019-54397-3)
Supplement: Supplementary file 1 — SUPPLEMENTARY TABLE 1 [file 41598_2019_54397_MOESM1_ESM.docx]

**Title:** **Effect of simultaneous presence of anti-blood group A/B and -HLA antibodies on clinical outcomes in kidney transplantation across positive crossmatch: a nationwide cohort study**

**Running title: ABO and crossmatch positive kidney transplants**

Hyunwook Kwon, Jee Yeon Kim, Dong Hyun Kim, Youngmin Ko, Ji Yoon Choi, Sung Shin, Joo Hee Jung, Young Hoon Kim*, Duck Jong Han, and The Korean Organ Transplantation Registry Study Group

**Corresponding author:** Young Hoon Kim, MD

Division of Kidney and Pancreas Transplantation, Department of Surgery, Asan Medical Center, University of Ulsan College of Medicine, 88, Olympic-ro 43-gil, Songpa-gu, Seoul 05505, Korea

Tel: +82-2-3010-3510; Fax: +82-2-3010-9207; E-mail: gskyh@amc.seoul.kr

**SUPPLEMENTARY TABLE 1:** Clinical outcomes at one year after ABO incompatible kidney transplantation

|  | ^a^ ABOi/XM- | ^a^ ABOi/XM+ | *P*-value |
| --- | --- | --- | --- |
| Number of patients | 296 (77.3) | 87 (22.7) |  |
| Overall rejection | 28 (9.5) | 22 (25.3) | <0.01 |
| ACR only | 10 (3.4) | 4 (4.6) | 0.59 |
| AMR with or without ACR | 18 (6.1) | 18 (20.7) | <0.01 |
| Overall graft survival | 279 (99.3) | 83 (95.4) | 0.02 |

Values are presented as numbers of patients (%).

Abbreviations: ACR, acute cellular rejection; AMR, Acute antibody-mediated rejection

^a^ Crossmatch-positive (XM+) defined as FCXM-positive and CDC XM-positive; ABOi, ABO incompatible
